# Genetic plasticity of the *Shigella* virulence plasmid is mediated by intra- and inter-molecular events between insertion sequences

**DOI:** 10.1371/journal.pgen.1007014

**Published:** 2017-09-25

**Authors:** Giulia Pilla, Gareth McVicker, Christoph M. Tang

**Affiliations:** Sir William Dunn School of Pathology, University of Oxford, Oxford, United Kingdom; Swiss Federal Institute of Technology Lausanne (EPFL), SWITZERLAND

## Abstract

Acquisition of a single copy, large virulence plasmid, pINV, led to the emergence of *Shigella* spp. from *Escherichia coli*. The plasmid encodes a Type III secretion system (T3SS) on a 30 kb pathogenicity island (PAI), and is maintained in a bacterial population through a series of toxin:antitoxin (TA) systems which mediate post-segregational killing (PSK). The T3SS imposes a significant cost on the bacterium, and strains which have lost the plasmid and/or genes encoding the T3SS grow faster than wild-type strains in the laboratory, and fail to bind the indicator dye Congo Red (CR). Our aim was to define the molecular events in *Shigella flexneri* that cause loss of Type III secretion (T3S), and to examine whether TA systems exert positional effects on pINV. During growth at 37°C, we found that deletions of regions of the plasmid including the PAI lead to the emergence of CR-negative colonies; deletions occur through intra-molecular recombination events between insertion sequences (ISs) flanking the PAI. Furthermore, by repositioning MvpAT (which belongs to the VapBC family of TA systems) near the PAI, we demonstrate that the location of this TA system alters the rearrangements that lead to loss of T3S, indicating that MvpAT acts both globally (by reducing loss of pINV through PSK) as well as locally (by preventing loss of adjacent sequences). During growth at environmental temperatures, we show for the first time that pINV spontaneously integrates into different sites in the chromosome, and this is mediated by inter-molecular events involving IS*1294*. Integration leads to reduced PAI gene expression and impaired secretion through the T3SS, while excision of pINV from the chromosome restores T3SS function. Therefore, pINV integration provides a reversible mechanism for *Shigella* to circumvent the metabolic burden imposed by pINV. Intra- and inter-molecular events between ISs, which are abundant in *Shigella* spp., mediate plasticity of *S*. *flexneri* pINV.

## Author summary

*Shigella flexneri* is the leading cause of bacillary dysentery worldwide. Key to its virulence is a large 210 kb single copy plasmid, pINV, which encodes a Type III Secretion System (T3SS) on a 30 kb pathogenicity island (PAI). When *S*. *flexneri* is grown on solid media containing Congo red (CR), virulent, T3SS-expressing colonies appear red (CR^+^). Colonies of bacteria are white and large (CR^-^) if they lose T3SS expression; thus, the T3SS imposes a significant metabolic burden on *S*. *flexneri*. Within the laboratory, spontaneous emergence of CR^-^ colonies is observed, but the molecular events responsible have not been defined. We characterised CR^-^ bacteria that arise during growth at 37°C and 21°C, and demonstrate that recombination between insertion sequences (ISs) on pINV results in loss of the PAI. Furthermore, we demonstrate that MvpAT, a member of the VapBC family of toxin:antitoxin systems encoded on pINV, is responsible for both plasmid maintenance through post-segregational killing, and retention of adjacent sequences. We show for the first time that ISs on the plasmid and chromosome mediate inter-molecular recombination events, resulting in spontaneous and reversible integration of pINV into the chromosome; following integration, T3SS expression is down-regulated. Therefore, integration/excision results in phenotypic heterogeneity that provides a bet-hedging strategy for *Shigella* to circumvent the metabolic burden associated with retaining virulence genes.

## Introduction

The genus *Shigella* is a major cause of diarrhoeal disease worldwide, and is responsible for around 188 million cases and 600,000 deaths each year [1, 2]. Most infections occur in low income countries where contaminated water and inadequate sanitation promote the transmission of the bacterium [2, 3]. *Shigella* is a human-specific pathogen that is divided into four species: *Shigella dysenteriae*, *Shigella flexneri*, *Shigella sonnei* and *Shigella boydii* [3]. Although the prevalence of each species depends on the geographic region, *S*. *flexneri* remains the leading cause of endemic shigellosis worldwide [2].

The four species of *Shigella* have emerged from *Escherichia coli* following the acquisition of a large plasmid, pINV, a 213 kb element that is essential for virulence [4]. pINV is a single copy, non-conjugative element that consists of a patchwork of pathogenesis-associated and plasmid maintenance genes, separated by regions of repeated sequences such as insertion sequence (IS) elements [5]. Indeed, ISs are highly abundant in *S*. *flexneri*, and account for 53% of pINV-encoded genes and 6.7% of all chromosomal sequence [5, 6].

Genes present on pINV enable the bacterium to invade intestinal epithelial cells, escape into the host cell cytosol, undergo cell-to-cell spread, and induce pyroptosis in macrophages [3, 7, 8]. Most of the virulence genes on pINV are located in a 30 kb pathogenicity island (PAI), which encodes components of a Type III Secretion System (T3SS), a molecular syringe that delivers bacterial effector proteins into the host cytoplasm [5, 9], with most secreted effectors also encoded by genes in the PAI. Expression of the T3SS is highly regulated and responds to specific environmental cues such as temperature [10], pH [11], osmolarity [10], oxygen [12], and iron concentrations [13]. Temperature is a key signal for *Shigella* [14], as it distinguishes between free-living and host-associated environments. The *Shigella* T3SS is activated at temperatures found in the gastrointestinal tract [14]; a rise in temperature to 37°C relieves H-NS repression of the pINV-encoded regulator VirF [15]. In turn, VirF activates the expression of another regulator, VirB, which is encoded on the PAI and controls expression of genes for the T3SS and its effectors [16].

For any single copy plasmid, its replication must be matched with the division of the chromosome, and active partitioning systems are needed to ensure that each daughter cell receives a copy of the plasmid on division. Furthermore plasmids can be maintained in bacterial populations through post-segregational killing (PSK) mechanisms, typically consisting of toxin:antitoxin (TA) systems which eliminate cells lacking a plasmid after division.

*S*. *flexneri* pINV possesses specific systems to prevent plasmid loss. To date, two partitioning systems, ParAB and StbAB, have been identified by sequence analysis [5], and three functional TA systems, MvpAT, CcdAB and GmvAT have been characterised in more detail [17–19]. Type II TA systems, such those found on pINV, are composed of genes encoding a toxic protein and a protein antidote. In general, the toxin is more stable than the antitoxin, with the antitoxin specifically degraded by proteases belonging to Lon or Clp families [20]. Typically, the antitoxin is produced at higher levels so that once degraded, it is rapidly replenished [21]. In the presence of the plasmid, the antitoxin counteracts the activity of the toxin, preventing cell death. However, once the plasmid is lost, the relatively unstable antitoxin is degraded and no longer replaced, leaving the toxin free to arrest cell growth, resulting in PSK [22]. MvpAT is the most characterized TA system on pINV and belongs to the VapBC family of TA systems [17–19]. The toxin MvpT is a site-specific endonuclease that stalls translation by cleaving tRNA^fMET^ [23]. MvpAT is essential for pINV maintenance at 37°C, while GmvAT confers pINV stability at environmental temperatures [19]. However, aside from the influence of temperature, the need for multiple TA systems on pINV and other plasmids remains unclear.

Colonies of virulent *S*. *flexneri* expressing a T3SS bind Congo red (CR) when grown on solid media containing this dye, giving rise to a CR^+^ phenotype [24]. As the T3SS is specifically expressed at 37°C [10, 25], binding to CR is only evident for colonies cultured at this temperature. In the laboratory, *S*. *flexneri* can spontaneously lose expression of its T3SS, resulting in white, avirulent colonies (CR^-^ phenotype) [26]. Of note, expression of the T3SS represents a high metabolic burden for *Shigella*, evident from the higher growth rate of CR^-^ strains compared with CR^+^ bacteria at 37°C [26, 27]. Although CR binding is widely used to distinguish between virulent and non-virulent *Shigella* [14], the molecular events that lead to *S*. *flexneri* becoming CR^-^ have not been characterised, although examples of segregational instability (*i*.*e*. loss of the entire plasmid), or structural instability (*i*.*e*. undefined deletions and/or rearrangements of pINV) have been described [14, 26–28].

Our aim was to define the genetic events that underlie the plasticity of pINV leading to the appearance of CR^-^ colonies during growth of *S*. *flexneri* at host and environmental temperatures. The majority of events occurring at 37°C result from structural instability of pINV, resulting in loss of the T3SS following intra-molecular events between ISs flanking the PAI. We also show that the TA system *mvpAT* not only contributes to segregational stability of pINV as described previously [19, 29], but also exerts local effects and prevents the loss of adjacent sequences. During growth at 21°C, we show for the first time that pINV spontaneously integrates into the chromosome. Chromosomal integration of *S*. *flexneri* pINV has been described previously [30], but only following exposure of bacteria with curing agents, or introduction of an incompatible plasmid. Furthermore, we observed that spontaneous integration occurs by inter-molecular events between copies of IS*1294* present on pINV and the chromosome, and leads to reduced expression of the T3SS regulatory cascade (*virF* and *virB*); CR^+^ revertants in which the plasmid had excised were recovered from strains with pINV integration. Therefore, integration provides a reversible mechanism for expression of the T3SS. Our findings highlight the importance of ISs in remodelling of *S*. *flexneri* pINV, and provide a framework for understanding changes in plasmid behaviour that influence the evolution and maintenance of virulence in this important human pathogen.

## Results

### MvpAT contributes to retention of pINV at 37°C

To examine the impact of MvpAT on the nature of pINV instability in *S*. *flexneri*, we introduced a point mutation into *mvpT* (*mvpT*^D7A^) to abolish its activity while leaving *mvpAT* in its original position on pINV [23] with *cat* marker downstream of *mvpAT* for selection; the strain was designated native *mvpAT*^D7A^, with “native” referring to its location on pINV. We also constructed an isogenic strain, native *mvp*^WT^, with the resistance marker in the same location, but downstream of a wild-type copy of *mvpAT*. The emergence of CR^-^ colonies from *S*. *flexneri* M90T, native *mvp*^D7A^ and native *mvp*^WT^ was assessed by measuring the proportion of CR^-^ colonies after approximately 50 generations of growth at 37°C, the temperature of the human intestinal tract.

**Fig 1:**
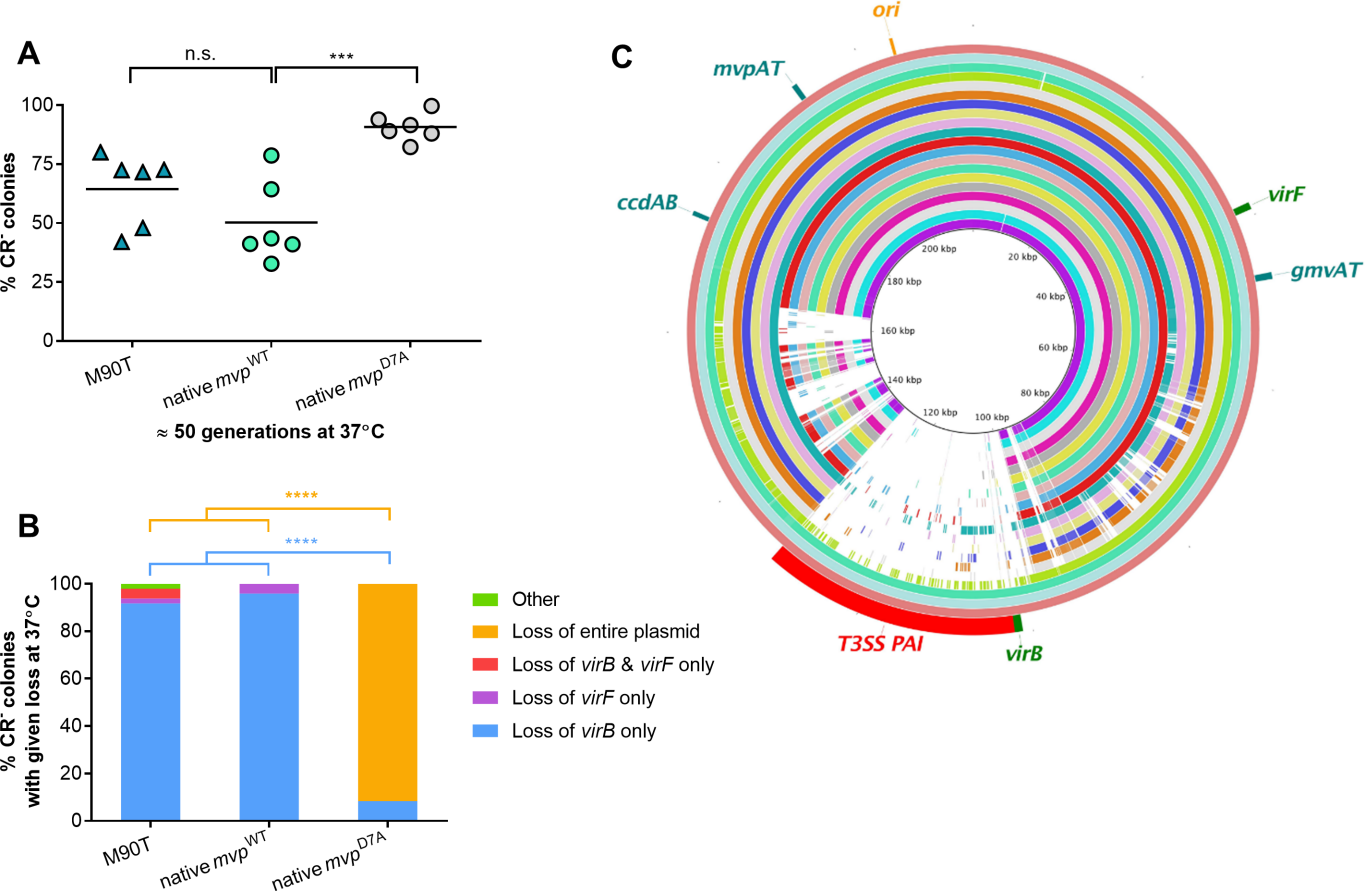
Emergence of CR^-^ colonies in *S*. *flexneri* results from loss of the PAI at 37°C. (A) Proportion of CR^-^ colonies in *S*. *flexneri* M90T, native *mvp*^WT^ *and* native *mvp*^D7A^ after approximately 50 generations at 37°C. Solid line: mean (n = six biological replicates). (B) Percentage of CR^-^ colonies lacking specified virulence-related genes. Loss of the entire plasmid is inferred by loss of the origin of replication; “others” refers to CR^-^ colonies that contain *virB*, *virF* and the origin of replication. Eight independent CR^-^ colonies obtained from native *mvp*^WT^ grown in six biological repeats (total 48 colonies) were analysed by multiplex PCR. ***, *p* ≤0.001; ****, *p* ≤0.0001; n.s., not significant; values analysed with one-way ANOVA, Tukey multiple comparisons test. (C) BLAST Ring Image Generator (BRIG 0.95 and BLASTN v2.2.29) alignment of plasmid sequences from twenty CR^-^ colonies that had emerged from native *mvp*^WT^ at 37°C; each ring represents the plasmid from an independent CR^-^ colony. *S*. *flexneri* M90T pWR100 (inner black ring) shown as the reference.

There was no significant difference in the number of CR^-^ bacteria arising from native *mvp*^WT^ and wild-type *S*. *flexneri* M90T, demonstrating that introduction of the *cat* cassette has no impact on the rate of emergence of CR^-^ bacteria (Fig 1A). Furthermore, consistent with previous work [19, 26], inactivation of *mvpAT* resulted in a significant increase in the proportion of CR^-^ colonies after 50 generations, which reached almost 90% in native *mvp*^D7A^; the average loss of CR binding did not exceed 60% of colonies of M90T or native *mvp*^WT^ following the same number of generations (native *mvp*^WT^ *vs*. native *mvp*^D7A^, *p* <0.001, Fig 1A).

Next we assessed whether MvpAT has any effect on the nature of pINV instability. Eight CR^-^ colonies arising from native *mvp*^WT^ or native *mvp*^D7A^ were isolated on six separate occasions (*i*.*e*. a total of 48 colonies), and examined by PCR for the presence of *virB*, *virF*, and the *ori* on pINV, and *hns* as a chromosomal control (Fig 1B). Loss of either *virB* and *virF* is sufficient to render *Shigella* CR^-^ [26], and *virB* is located within the T3SS PAI [5]. Amplification of the replication origin (*ori*) was used to monitor the presence of pINV. We found that *virB* was the only gene not amplified by multiplex PCR from 92% of CR^-^ colonies emerging from *S*. *flexneri* M90T (Fig 1B). A similar result was obtained for native *mvp*^WT^, demonstrating that the presence of *cat* downstream of *mvpAT* does not affect the nature of emerging CR^-^ colonies (loss of *virB* in *S*. *flexneri* M90T *vs*. native *mvp*^WT^, *p* >0.9999). However, the profile of CR^-^ bacteria arising from native *mvp*^D7A^ was distinct from *S*. *flexneri* M90T and native *mvp*^WT^; loss of the entire plasmid was the major cause of loss of CR binding in native *mvp*^D7A^, accounting 92% of CR^-^ colonies (Fig 1B; native *mvp*^D7A^ vs. native *mvp*^WT^, *p* <0.0001); loss of *virB* alone (as detected by multiplex PCR) was associated with only 8% of CR^-^ colonies in native *mvp*^D7A^.

Taken together, these data confirm that *mvpAT* is fundamental for plasmid stability at 37°C [19, 29] and contributes to the maintenance of the entire plasmid, consistent with its role in PSK. Inactivation of MvpAT by introducing a non-toxic allele of *mvpT* leads a dramatic increase in the CR^-^ phenotype, mainly due loss of pINV (*i*.*e*. segregational instability). However, in contrast to a previous study of a different *S*. *flexneri* strain [26], we found that in wild-type M90T *S*. *flexneri* CR^-^ bacteria mostly arise following deletions involving the PAI rather than loss of the entire plasmid.

### Spontaneous loss of the T3SS PAI occurs by least four distinct routes

To further characterise the molecular events responsible for the emergence of CR^-^ *S*. *flexneri*, we performed whole genome sequencing of 20 CR^-^ colonies independently derived from native *mvp*^WT^ at 37°C (Fig 1C); plasmid sequences were aligned with BLAST Ring Image Generator with pINV from *S*. *flexneri* M90T as the reference [9]. Results confirmed that CR binding is mainly lost at 37°C following loss of the T3SS PAI and not loss of pINV (Fig 1C). Out of 20 CR^-^ colonies, 17 lacked sequences unique to the PAI, with the remaining three displaying an intact plasmid sequence (Fig 1C); the regions of homology within the PAI in the 17 strains correspond to sequences also present elsewhere on pINV or on the chromosome, such as IS elements [5], so represent an alignment artefact.

**Fig 2:**
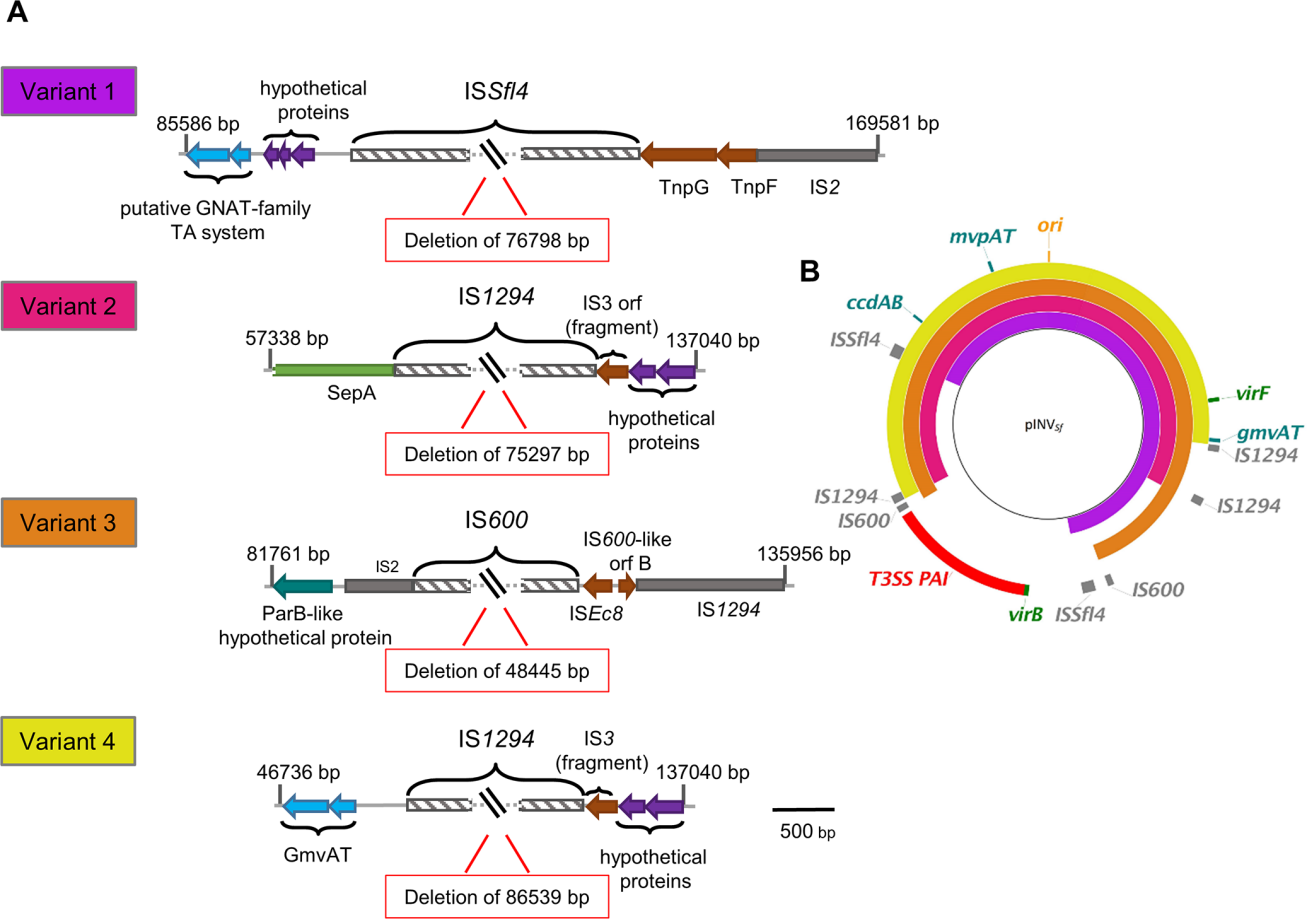
Loss of the T3SS PAI occurs by least four distinct routes. (A) Recombination sites leading to loss of the PAI in CR^-^ colonies showing the sizes of deleted fragments and ISs (hatched boxes). ORFs are shown according to their predicted functions: (in cyan blue) TA systems; (in purple) hypothetical proteins; (in green) virulence-associated proteins; (in grey) homologues of ISs; (in brown) IS components; (in turquoise) homologues of proteins involved in plasmid partitioning; (hatched boxes) ISs involved in the deletion. (B) Alignment of the four PAI deletions with pWR100 (inner black ring). Image created using BRIG v0.95 and BLAST v2.2.29.

To define the sequences mediating loss of the PAI, we performed PCR to amplify regions flanking the deletions (Fig 2 and S3 Table for primer sequences), and analysed the products by restriction enzyme digestion and sequencing. We identified four events leading to loss of the PAI, each involving a pair of homologous ISs (Fig 2A). The most frequent deletion (Variant 1) occurred in 11 out of 17 CR^-^ colonies and involved two copies of IS*Sfl4*, spanning positions 87,663–90,277 bp and 164,461–167,075 bp of pINV [9]. The second most frequent deletion event (Variant 2) occurred in four of colonies and included two copies of IS*1294*, located at positions 58,970–60,658 bp and 134,268–135,956 bp. Variant 3 and 4 each accounted for a single CR^-^ colony and employed copies of IS*600* (83,866–85,129 bp and 132,311–133,574 bp) and IS*1294* (48,065–49,417 bp and 134,268–135,956 bp), respectively. Taken together these results demonstrate that ISs on pINV are “hot spots” for recombination, resulting in deletion of different regions of the plasmid that include the PAI (S1 Table).

### MvpAT governs loss of local sequences

**Fig 3:**
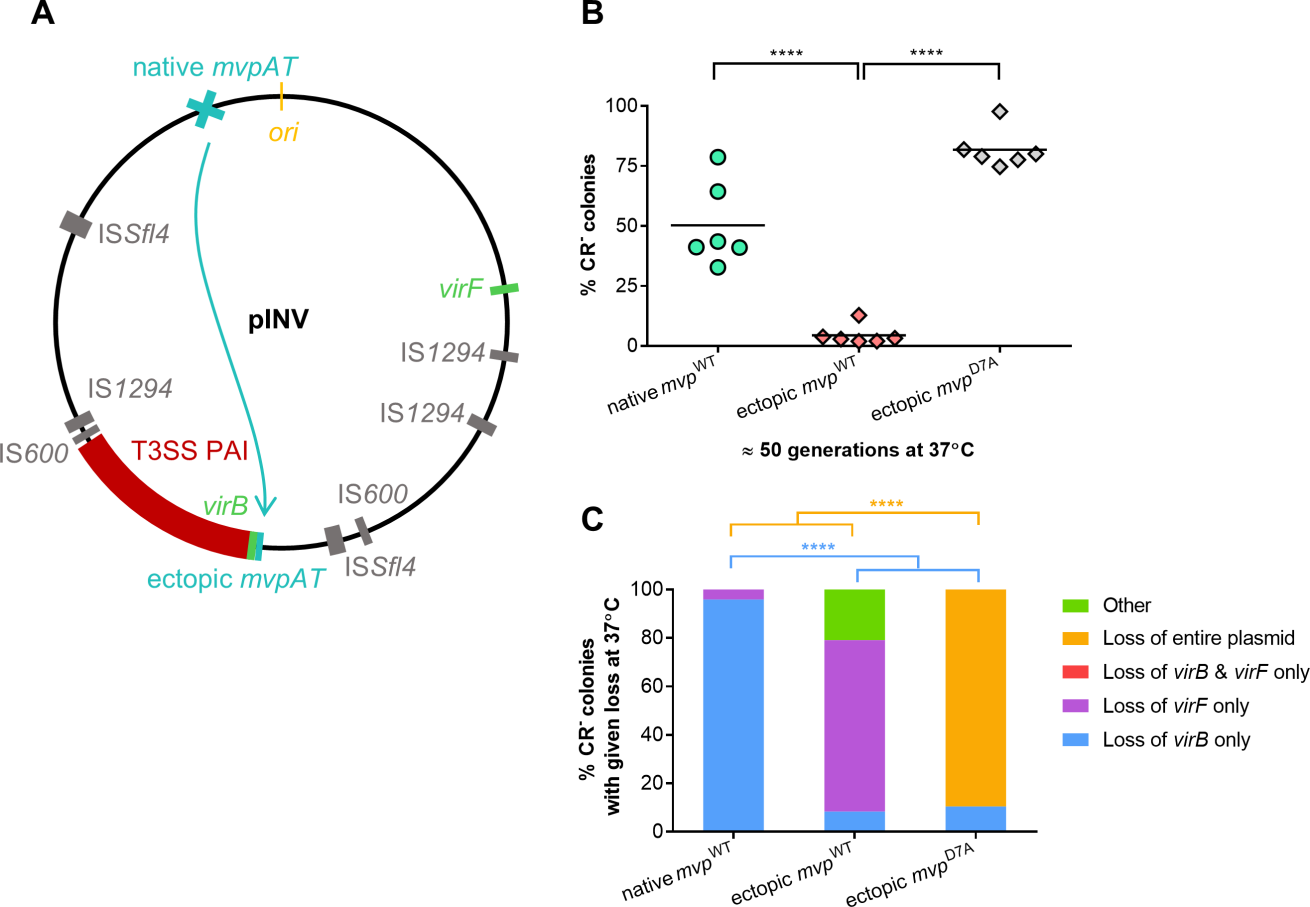
The position of *mvpAT* influences loss of CR binding. (A) Schematic representation of the repositioning of *mvpAT* near the T3SS PAI in ectopic *mvp* strains. (B) Proportion of CR^-^ colonies in *S*. *flexneri* native *mvp*^WT^ (reproduced from Fig 1A for statistical comparison), native, ectopic *mvp*^WT^ and ectopic *mvp*^D7A^ after approximately 50 generations growth at 37°C. Solid line: mean of six biological replicates. (C) Multiplex PCR analysis was performed as described for Fig 1B. Results are shown as mean (n = six biological replicates). ****, *p* ≤ 0.0001; values analysed with one-way ANOVA, Tukey multiple comparisons test.

The reason why *S*. *flexneri* pINV has three functional TA systems is unclear [19]. As we found that distinct regions on pINV can be lost by IS-mediated recombination, we hypothesised that the presence of multiple TA systems could be due to a positional effect of these elements on plasmid dynamics. To assess whether the position of *mvpAT* influences plasmid stability, we deleted *mvpAT* from pINV and introduced either a wild-type or a non-functional version (*mvp*^D7A^) of *mvpAT* adjacent to the T3SS PAI, at nt. 100,792 of pINV [9], generating ectopic *mvp*^WT^ and ectopic *mvp*^D7A^, respectively (Fig 3A). Similar to native *mvp*^D7A^, when the non-functional allele was introduced into the ectopic site, the inactivity of MvpAT led to a large population of CR^-^ bacteria emerging after 50 generations (~ 80% of the total population, Fig 3B, ectopic *mvp*^WT^ *vs*. ectopic *mvp*^D7A^, *p* <0.0001), most of which resulted from loss of the entire plasmid. This demonstrates that MvpAT contributes to retention of pINV, irrespective of its position on the plasmid.

However, there was a dramatic reduction in the number of CR^-^ bacteria emerging from ectopic *mvp*^WT^ compared with native *mvp*^WT^ at 37°C (Fig 3B, *p* <0.0001). Indeed, over approximately 50 generations, the proportion of CR^-^ colonies in ectopic *mvp*^WT^ did not exceed 5%, compared with ~ 50% for native *mvp*^WT^ (Fig 3B). Furthermore, there was a striking change in the events leading to CR^-^ colonies when *mvpAT* is positioned next to the PAI. By multiplex PCR, loss of *virB* represented a significantly lower proportion of CR^-^ colonies emerging from ectopic *mvp*^WT^ compared with native *mvp*^WT^ (Fig 3C, *p* <0.0001), and accounted for only 8% of CR^-^ colonies emerging from ectopic *mvp*^WT^. Instead, loss of *virF* was the most frequent event, accounting for 71% of CR^-^ derivatives (ectopic *mvp*^WT^ *vs*. native *mvp*^WT^, p<0.0001). However, differences in the proportion of genetic changes leading to loss of CR binding observed for native *mvp*^WT^ and ectopic *mvp*^WT^ need to be considered in the context of the rate of emergence of CR^-^ colonies in these strains. The absolute values for the loss of *virF* in ectopic *mvp*^WT^ are comparable to those observed for native *mvp*^WT^ (3.55% and 2% of all colonies, respectively). Thus, the position of *mvp* on pINV affects loss of *virB* but not loss of *virF*, demonstrating that *mvp* influences structural plasmid stability by acting to retain nearby sequences.

### IS-mediated deletions and plasmid insertion lead to CR^-^ colonies at environmental temperatures

**Fig 4:**
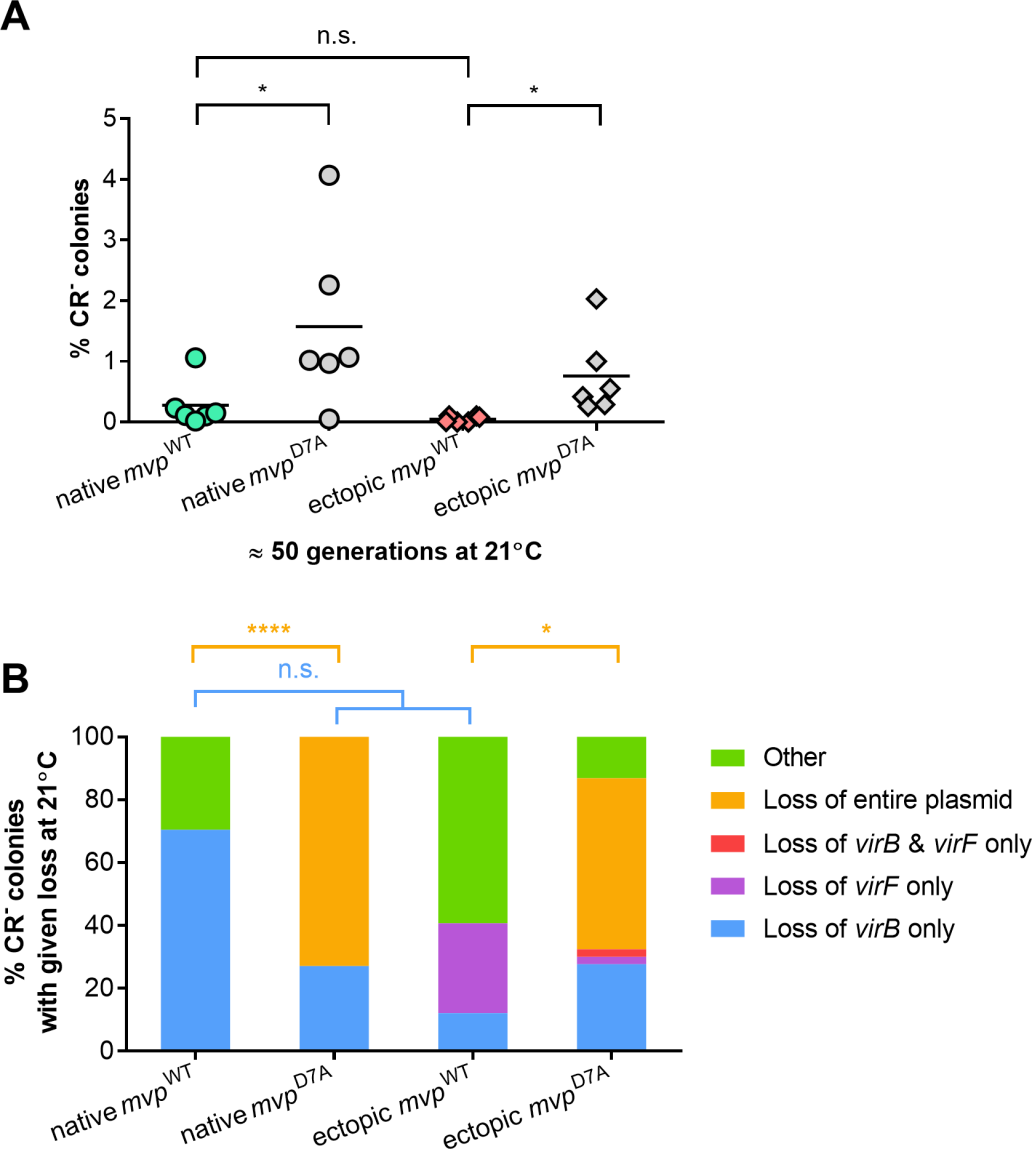
Characterization of CR^-^ colonies emerging at 21°C. (A) Proportion of CR^-^ colonies in *S*. *flexneri* native *mvp*^WT^, native *mvp*^D7A^, ectopic *mvp*^WT^ and ectopic *mvp*^D7A^ after approximately 50 generations at 21°C. Solid line: mean of six biological replicates. (B) Multiplex PCR analysis was performed as described for Fig 1B. Results are shown as mean (n = six biological replicates). *, *p* ≤ 0.05; ****, *p* ≤ 0.0001; n.s., not significant; values analysed with one-way ANOVA, Tukey multiple comparisons test.

We also examined the emergence of CR^-^ colonies at 21°C, the temperature the bacterium faces in the external environment during host-to-host transmission. Bacteria were grown in liquid media at 21°C for approximately 50 generations, then aliquots were plated on solid media containing CR and incubated at 37°C, as the CR binding is visible at 37°C but not at 21°C. We found previously that, although there is no difference in the stability of pINV in *S*. *flexneri* at 37°C and at 21°C [19], a large population of CR^-^ bacteria emerges at the higher temperature from wild-type *S*. *flexneri* (Fig 1) because of the growth advantage of CR^-^ colonies at 37°C. As expected, when *S*. *flexneri* M90T was grown at 21°C, fewer CR^-^ colonies emerged after 50 generations than at 37°C, and accounted for approximately 2% of the total population (Fig 4A); again results for native *mvp*^WT^ were indistinguishable from *S*. *flexneri* M90T, confirming that the presence of the *cat* cassette does not affect plasmid stability (S1 Fig). Furthermore, there was no significant difference in the number of CR^-^ bacteria emerging from ectopic *mvp*^WT^ and native *mvp*^WT^ (p = 0.6603). However, there was a slight but significant increase in the proportion of CR^-^ colonies emerging from strains with the inactive *mvp*^D7A^ allele compared with control strains (Fig 4A, native *mvp*^WT^ *vs*. native *mvp*^D7A^, p = 0.0449; ectopic *mvp*^WT^ *vs*. ectopic *mvp*^D7A^, p = 0.0397), consistent with our previous findings [19] that MvpAT possesses some residual activity at environmental temperatures.

Next, we examined the nature of CR^-^ bacteria emerging at 21°C by multiplex PCR (Fig 4B). Similar to 37°C, loss of *virB* still prevailed in native *mvp*^WT^, and accounted for 80% of CR^-^ colonies. Furthermore, the majority of CR^-^ bacteria derived from native *mvp*^D7A^ and ectopic *mvp*^D7A^ had lost pINV, accounting for 73% and 55% of CR^-^ colonies, respectively (Fig 4B; native *mvp*^WT^ *vs*. native *mvp*^D7A^, *p* <0.0001; ectopic *mvp*^WT^ *vs*. ectopic *mvp*^D7A^, p = 0.0454), demonstrating that MvpAT prevents plasmid loss at 21°C as well as 37°C. However, we found that a considerable number of CR^-^ colonies emerging at 21°C from strains from wild-type *mvpAT* retained *virB*, *virF*, and *ori* (Fig 4B). More than 25% and 50% of all CR^-^ colonies derived from native *mvp*^WT^ and ectopic *mvp*^WT^, respectively, harboured all three pINV genes showing that alternative mechanisms might be responsible for the emergence of CR^-^ bacteria at 21°C**.** Illumina sequencing of nine CR^-^ colonies that emerged independently at 21°C from native *mvp*^WT^ and ectopic *mvp*^WT^ confirmed that these strains harboured an intact plasmid with no detectable single nucleotide polymorphisms (S2 Fig).

### pINV reversibly integrates into the chromosome

**Fig 5:**
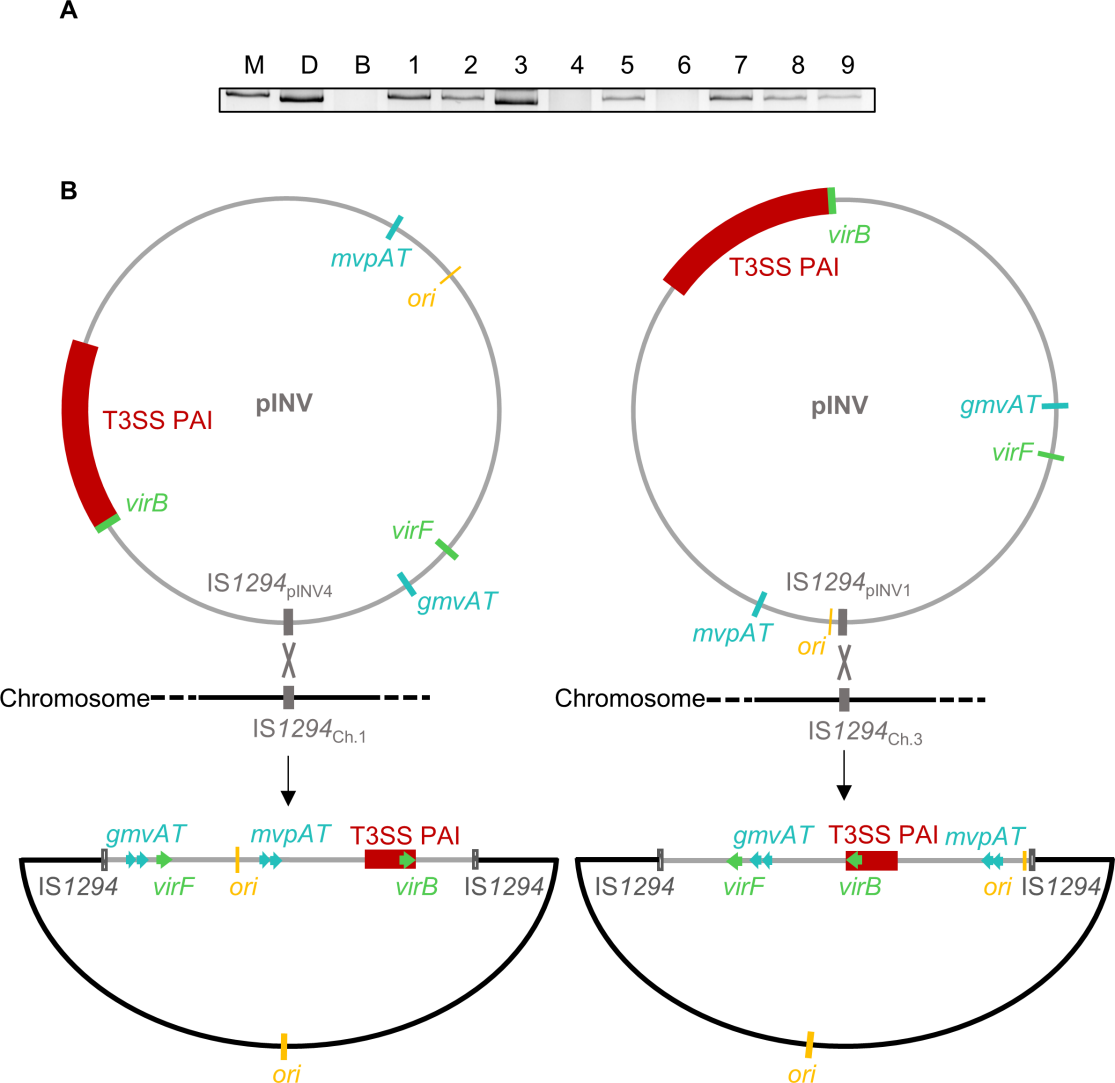
Chromosomal integration of pINV occurs *via* distinct copies of IS*1294*. (A) Agarose gel electrophoresis of pINV DNA purified from nine independent CR^-^ colonies (lanes 1–9) emerging from native *mvp*^WT^ at 21°C that retained all the virulence-related genes tested by multiplex PCR. Lanes: (lane labelled M) M90T, (B) BS176, (D) T3SS PAI-deleted M90T. (B) Schematic representation of chromosomal pINV integration in isolate 4 (left) and isolate 6 (right). The chromosomal and plasmid origins of replication are shown in yellow. ORFs are shown with the same colour coding as in Fig 2.

To identify the mechanisms by which CR^-^ bacteria emerge at 21°C, we analysed the nine CR^-^ colonies with intact pINV sequence which arose from native *mvp*^WT^ (Fig 4B). It has previously been shown that pINV of *S*. *flexneri* M90T can integrate into the host chromosome [30], but only following exposure of bacteria to curing agents, or selecting for acquisition of a plasmid with an *ori* which is incompatible with pINV. Therefore, we hypothesised that plasmid integration could occur spontaneously, so analysed plasmid DNA extracted from the nine CR^-^ isolates. Results demonstrate that a distinct band for pINV was missing in two out of nine CR^-^ strains (Fig 5A, isolates 4 and 6). As previous Illumina sequence analysis confirmed that neither strain had lost any pINV sequence (S2 Fig), isolates 4 and 6 were subjected to PacBio sequencing. In both strains, we found an intact copy of pINV integrated into the chromosome *via* different copies of IS*1294* (Fig 5B and S3 Fig); there are six copies of IS*1294* on the plasmid and three copies on the chromosome. Integration in strain 4 involved the chromosomal copy of IS*1294* at position 1,615,894–1,617,578 bp [31] and pINV IS*1294* at 58,970–60,658 bp [9] (*i*.*e*. IS*1294*_pINV4_ and IS*1294*_Ch.1_, respectively, S3 Fig); for isolate 6, chromosomal IS*1294* at position 1,872,287–1,874,073 bp [31] and plasmid IS*1294* at position 205,186–206,599 bp are involved (IS*1294*_pINV1_ and IS*1294*_Ch.3_, respectively, S3 Fig) [9].

**Fig 6:**
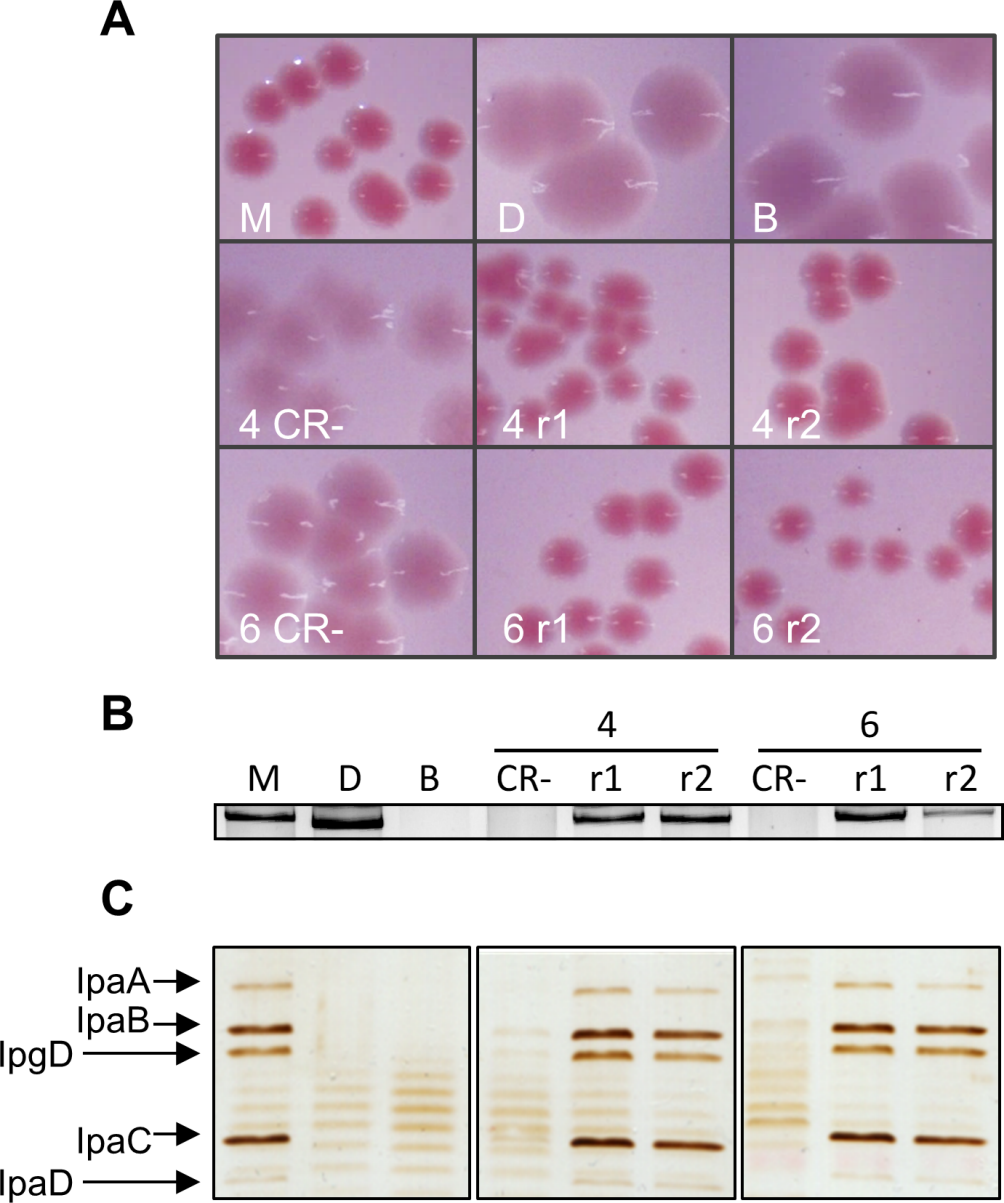
Excision of pINV restores type III secretion. (A) CR-TSA plates of strains grown at 37°C overnight: M90T (M), BS176 (B), T3SS PAI-deleted M90T (D), isolate 4 and its revertants (middle row), and isolate 6 and its revertants (lower row). (B) Agarose gel electrophoresis of purified plasmid DNA from control strains as for Panel A and plasmid-integrated isolates 4 and 6 (CR^-^), and two revertants (r1 and r2) derived from isolates 4 and 6. (C) Secretion through the T3SS induced by CR. Legend as for panel B. The bands corresponding to secreted effectors are indicated.

Furthermore, we investigated whether integration of pINV is a reversible event. We examined isolate 4 and 6 for the emergence of CR^+^ colonies, as phenotypic evidence for plasmid excision. The isolates were grown overnight in liquid media at 37°C and plated to solid media containing CR. CR^+^ colonies emerged from both isolates (Fig 6A), and we analysed plasmid DNA from two independent CR^+^ revertants (Fig 6B). Results confirm that pINV not only integrates into the host chromosome, but can also subsequently excise, restoring the CR^+^ phenotype (Fig 6A).

### Chromosomal integration of pINV down-regulates expression of PAI genes

Next we examined the activity of the T3SS in isolates 4 and 6, and their corresponding revertants. Bacteria were grown to exponential phase at 37°C in liquid media and exposed to CR to induce secretion through the T3SS [32]. Silver staining of secreted proteins demonstrates that both isolates 4 and 6 fail to secrete the T3SS effectors, IpaA, IpaB, IpaC, IpaD and IpgD (Fig 6C). In contrast, the revertants secrete T3SS effectors at levels similar to *S*. *flexneri* M90T (Fig 6C), indicating that integration and excision of pINV provides a reversible mechanism that controls T3SS activity.

**Fig 7:**
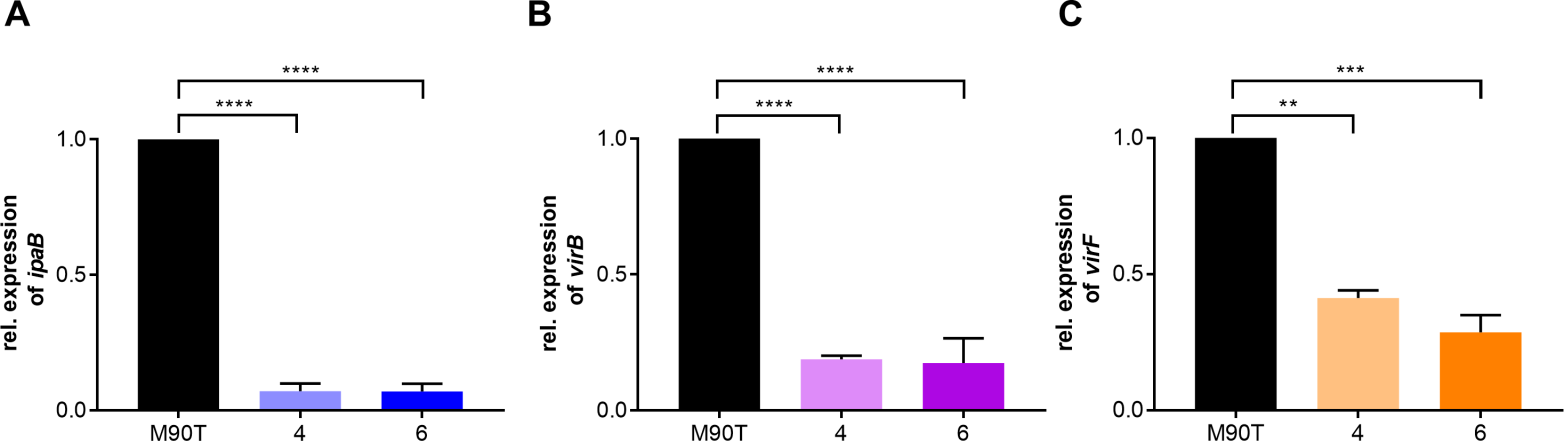
pINV integration results in downregulation of T3SS PAI gene expression. qRT-PCR analysis of *ipaB* (A), *virB* (B) and *virF* (C) in M90T, and isolates 4 and 6. Relative gene expression was determined by the 2^-ΔΔ^ method. Error bars show mean + S.E.M. of four biological replicates. **, *p* ≤ 0.01; ***, *p* ≤ 0.001 ****, *p* ≤ 0.0001. Values analysed with one-way ANOVA, Tukey multiple comparisons test.

To determine the mechanisms underlying the lack of secretion through the T3SS in the pINV-integrated isolates, we examined mRNA levels of *ipaB*, *virB* and *virF* in strains 4 and 6, and wild-type *S*. *flexneri* M90T. mRNA levels were measured by qRT-PCR in bacteria during exponential growth at 37°C, and results were normalized to the expression of the chromosomal gene, *polA* (Fig 7). Consistent with the secretion assays, mRNA levels of *ipaB* were significantly lower in the two strains with plasmid integration (Fig 7A, M90T *vs*. strain 4 or strain 6, *p* <0.0001). A similar statistically significant trend was shown for mRNA levels of *virB* (Fig 7B, M90T *vs*. strain 4 or strain 6, *p* <0.0001) and *virF* (Fig 7C, M90T *vs*. strain 4 *p* <0.01; M90T *vs*. strain 6, *p* <0.001), suggesting that the observed down-regulation of the T3SS PAI in plasmid-integrated isolates is correlated with reduced expression of *virB* and *virF*.

## Discussion

For many pathogenic bacteria, large plasmids are critical for their virulence and/or the spread of antimicrobial resistance [33–36]. While plasmids confer beneficial traits to bacteria in certain circumstances, they often impose a considerable metabolic cost on the host cell so have evolved dedicated mechanisms to ensure their maintenance within a bacterial population. Genetic plasticity is a fundamental feature of many large plasmids, and facilitates rearrangements that allow appropriate gene expression and the acquisition of novel traits such as antibiotic resistance, virulence or metabolic capabilities [37], facilitating adaptation to new ecological niches. *S*. *sonnei* pINV is an example of how genetic events contribute to the evolution of the plasmid, reflecting bacterial adaptation to new lifestyles [19]. For example, *S*. *sonnei* pINV has lost two TA systems and a partitioning system, and acquired an O-antigen gene cluster [19, 38], during its transition to a species undergoing predominant host-to-host transmission [19, 39].

Here we analysed the genetic changes associated with plasticity of pINV. The dynamic nature of *S*. *flexneri* pINV is a feature shared with plasmids in other *Shigella* spp., which contain a high proportion of ISs; ISs represent approximately 53% of the ORFs on pINV [5]. These elements have shaped the evolution of *Shigella* by acting as substrates for recombination, mediating inversions, translocations, insertions and deletions [37]. On the chromosome, similar processes have led to loss of co-linearity of genomes of four *Shigella* species, and gene loss associated with enhanced virulence [40, 41]. Nonetheless, the T3SS PAI and most other pINV encoded molecular effectors have been maintained in all *Shigella* spp., suggesting that selection pressure has preserved these sequences in disease-causing isolates.

We demonstrate that ISs influence the architecture of *Shigella* pINV and mediate a series of deletions that result in the loss of PAI-associated virulence genes and CR binding during growth in the laboratory. Others have found that the most frequent cause of loss of the CR^+^ phenotype during extended growth of *S*. *flexneri* 2a at 37°C is loss of the entire plasmid [26]. In contrast, we show that the predominant event leading to loss of CR binding in *S*. *flexneri* 5a M90T is deletion of the T3SS PAI (Fig 1), consistent with other studies [27, 28, 42]. This discrepancy could be explained by the different MvpAs in the strains; for example, MvpA in M90T has a single amino acid difference (Glu^70^ instead of His^70^) compared with the corresponding protein in the *S*. *flexneri* 2a strain used in previous work [26, 43]. However, the consequences of this difference in MvpAT are unknown. We found that IS*Sfl4* is the most common IS involved in PAI deletion (Fig 2), even though there are only two copies of this IS on pINV. The plasmid harbours multiple copies of other ISs which occupy a far greater proportion of the plasmid sequence than IS*Sfl4*. Therefore, the reason why this pair of ISs is prone to recombination is not clear. The frequency of recombination between pairs of ISs depends on several factors including the extent and length of homology, the coverage of sequence and local DNA topology (S1 Table). In fact, the percentage of coverage between the ISs involved in the less frequent deletions is lower than for the pair of ISs implicated in Variant 1 strains, which have the commonest rearrangement (S1 Table).

Similar to previous work [17, 19, 29], we found that MvpAT plays a critical role in segregational stability of pINV. Inactivation of this TA system led to increased loss of pINV at 37°C. We also demonstrated that the location of *mvpAT* is critical in governing plasmid dynamics at a local level; repositioning *mvpAT* to near the PAI dramatically reduced the loss of this region (Fig 3). The likely reason for this is ‘post-recombinational killing’ whereby recombination between flanking ISs causes loss of *mvpAT* and cell death in a manner analogous to PSK. To our knowledge, this is the first description of TA loci exerting local effects on plasmids, although they can prevent large-scale deletions of adjacent sequences on bacterial chromosomes [44, 45]. The localised effects of MvpAT and other TA systems might provide an explanation for the number and distribution of TA systems on pINV. Of note, *mvpAT* is located close to the *ori* of pINV in all *Shigella* species.

Following growth at 21°C, we identified CR^-^ strains emerging from *S*. *flexneri* that retained all plasmid genes. By analysis of this population of strains, we demonstrate that pINV can spontaneously integrate into the *Shigella* chromosome. Previous reports of chromosomal integration of *Shigella* pINV and the related plasmid from enteroinvasive *E*. *coli* have only followed exposing bacteria to curing agents (such as rifampicin) or by introducing other plasmids with pINV-incompatible replicons [30]. Following these artificial treatments, pINV usually integrated into *metB*, leading to methionine auxotrophy. In contrast, we found that chromosomal and plasmid copies of IS*1294* mediate intermolecular recombination, resulting in integration of pINV at different sites in the chromosome. At both chromosomal sites, integration led to loss of CR binding and prevented secretion through the T3SS (Fig 6B). Comparing pINV-integrated strains with the wild-type strain, integration was associated with reduced levels of mRNA for *virF* and *virB*, the two transcription factors involved in the activation cascade of T3SS PAI expression, and *ipaB*, which encodes a T3SS-secreted protein. We also demonstrate that integration is reversible and excision occurs during bacterial growth at 37°C (Fig 5).

The mechanisms responsible for reduced T3SS activity following pINV integration are unknown. H-NS is a transcriptional repressor of the genes in the PAI, which binds to the promoters of *virF* and *virB* in a temperature-dependent manner due to alterations in DNA topology [15]. When integration of pINV from *S*. *flexneri* and enteroinvasive *E*. *coli* was forced and occurred at *metB*, H-NS repression of the *virB* promoter was enhanced, probably due to alteration in the topology of pINV DNA when in the chromosome [46]. However, our qRT-PCR results showed that spontaneous integration *via* IS*1294* also affects *virF* expression (Fig 7C). The *E*. *coli* and *Salmonella* chromosomes are organised into topologically distinct regions, containing structured DNA macrodomains [47–49]. As the architecture of the chromosome is not homogenous and other ISs could also mediate integration, the exact site and orientation of pINV integration are likely to influence gene expression [50]. Further experiments are underway to define the precise mechanisms modulating changes in T3SS expression following spontaneous integration of pINV through ISs.

Other examples of the integration of large plasmids in pathogens include the *Salmonella typhimurium* virulence plasmid [51]. Following plasmid integration, *Salmonella* becomes susceptible to complement-mediated killing probably through down-regulation of gene expression. Serum resistance can be restored by introduction of an autonomous plasmid harbouring a copy of *rsk*, a plasmid gene responsible for resistance to complement [51].

Our findings define the molecular rearrangements that affect *S*. *flexneri* pINV, and highlight the importance of IS elements in processes that are fundamental for plasmid remodelling and evolution. We found that intramolecular events between ISs on pINV lead to the emergence of CR^-^ bacteria lacking the T3SS PAI during growth of *S*. *flexneri*. CR^-^ bacteria have a significant growth advantage in rich media at 37°C compared with CR^+^ bacteria, and quickly proliferate and dominate a bacterial population [27]. While the loss of a T3SS could hinder survival of *Shigella* within the intestine, the fitness advantage upon deletion of the PAI could prove advantageous for *Shigella* outside the host. Indeed, a significant proportion of *Shigella* isolated from aquatic environments have lost key virulence genes on pINV [52], consistent with IS-mediated events. However, deletion of the PAI T3SS and loss of virulence, is a one-way process, that can only be reversed by acquisition of a new plasmid. Although *S*. *flexneri* pINV has an origin of transfer, the plasmid is incapable of self-mobilisation [53]. Therefore, IS mediated deletions causing PAI loss are likely to be deleterious in the longer term as they will render the bacterium non-invasive.

In contrast, pINV integration follows an inter-molecular event that is completely reversible. We recovered virulent revertants in which pINV had excised and the T3SS was functional. Therefore, plasmid integration *via* ISs offers *S*. *flexneri* an alternative strategy to maintain the plasmid within dividing bacteria while circumventing the fitness costs imposed by expression of the T3SS. pINV integration/excision leads to bi-stable expression of virulence genes by *Shigella*, which provides a mechanism for the avoidance of host responses against immunogenic T3SS components [54], and for phenotypic heterogeneity without genetic loss which would promote the evolutionary stability of virulent *Shigella*.

## Materials and methods

### Strains and growth media

Bacterial strains used for this study are shown in S2 Table. *S*. *flexneri* was grown in Tryptic Soy Broth (TSB; Sigma Aldrich, St. Louis, USA) or on Tryptic Soy solid media containing 1.5% (w/v) agar (Oxoid, Basingstoke, UK) (TSA). Chloramphenicol was used as appropriate at a final concentration of 20 μg/mL. Congo red (Sigma Aldrich, St. Louis, USA) was added to TSA media at a final concentration of 0.01% w/v to make CR-TSA plates.

### Construction of strains

DNA constructs were ligated into pUC19 using the NEBuilder HiFi master mix (New England Biolabs, NEB, Ipswich, MA), and PCR products were generated using primers shown in S3 Table. Resulting plasmids were transformed into *E*. *coli* DH5α and linear DNA constructs were amplified by PCR using plasmids as the template. Lambda Red recombination [55, 56] was used to introduce changes into pINV in *S*. *flexneri* M90T. Approximately 1 kb of homologous flanking sequence was used to allow integration into the plasmid. For strains native *mvpAT*^D7A^ and ectopic *mvpAT*^D7A^, a point mutation was introduced into *mvpAT* by site-directed mutagenesis (S3 Table). Mutations were then transduced into *S*. *flexneri* using P1*vir* [19]. In ectopic *mvpAT*^WT^ and ectopic *mvpAT*^D7A^, the native *mvpAT* locus was deleted and either the wild-type or the mutated *mvpAT* were positioned between *virB* and *ipaJ* at nt. 100,792 on pINV [9].

### CR binding assays

*S*. *flexneri* was grown on CR-TSA plates overnight at the selected temperature to obtain single colonies. Three CR^+^ colonies from each strain were re-suspended in 5mL TSB and incubated at 30°C, 180 r.p.m.; there is no detectable growth rate difference between CR^+^ and CR^-^ bacteria at this temperature [19], so CR^-^ bacteria do not outcompete CR^+^ bacteria. After this initial overnight growth, cultures were grown for approximately 50 generations at either 37°C or 21°C by sub-culture. Samples were diluted in PBS and plated onto CR-TSA, and incubated overnight at 37°C before CR^+^ and CR^-^ colonies were counted.

### Multiplex PCR for genes on pINV

Multiplex PCR was performed with primers (S3 Table) to amplify *virF*, *virB*, *mvpAT*, and *ori* to generate products of distinct sizes; *hns*, a chromosomal gene, was included as a control (S4 Fig). Reactions included Taq polymerase (Sigma Aldrich, St. Louis, USA) with an annealing temperature of 51.2°C and extension time of 1.5 min. Eight CR^-^ colonies emerging on six independent occasions were analysed by multiplex PCR for each strain. Statistical analysis of results was performed using two-way ANOVA with Tukey’s post test comparison, evaluating loss of a single locus alone or in pairwise comparison with other loci.

### Genome sequencing

For Illumina sequencing, genomic DNA was purified from bacteria grown overnight in 5 mL TSB using Charge Switch gDNA Mini Bacteria Kit (Thermo Fisher Scientific, MA, USA). Colonies were isolated after plating at 37°C or 21°C and grown at 37°C for 16 hrs or 21°C for 24 hrs, respectively, before DNA isolation. DNA was sequenced at The Wellcome Trust Centre for Human Genetics at the University of Oxford. Sequence data were analysed using Snap Gene for identifying single nucleotide polymorphisms and deletions, while BLAST Ring Image Generator (BRIG) was employed to align plasmid sequences [57]. For both approaches, *S*. *flexneri* M90T was used as the reference. For PacBio sequencing [58], DNA was recovered from a loop of colonies using Wizard Genomic DNA Purification kit (Promega, WI, USA). Sequencing was performed at The Earlham Institute, Norwich.

### Purification and visualization of pINV, and protein secretion

Isolates were plated on CR-TSA from 15% glycerol stocks, and incubated at 37°C overnight. One colony from each isolate was streaked again onto CR-TSA and grown as above. A loop of each solid culture was then processed for pINV extraction as previously described [59]. DNA samples were run on 0.7% agarose gel at 1 V/cm voltage for approximately 16 hrs.

To activate secretion through the T3SS, bacteria were grown overnight at 37°C in TSB at 180 r.p.m., then sub-cultured to an OD_600_ of 0.05 into 10mL fresh TSB grown in the same conditions until OD_600_ ~ 1 was reached. Cultures were centrifuged and pellets were resuspended in PBS to an OD_600_ ~ 5. T3SS secretion was induced by adding Congo red (final concentration, 0.02% w/v) followed by incubation at 37°C for 15 minutes. Bacteria were pelleted and supernatants containing secreted proteins were boiled in an equal volume of 2x SDS-PAGE loading buffer (0.1M Tris-HCl ph 6.8, 1.5% SDS, 20% glycerol, 25mM EDTA, 2% β-mercaptoethanol, 150 μg/mL bromophenol blue) at a 1:1 dilution before loaded on 10% SDS-PAGE gels. After electrophoresis, gels were silver stained using the SilverXpress Kit (Invitrogen LC6100) following manufacter’s protocol.

### RNA extraction and quantitative real-time PCR

Bacteria were grown at 37°C overnight in TSB then sub-cultured to OD600 = 0.05 into 25mL fresh TSB and grown at 37°C until the OD_600_ reached ~ 1. A 20mL aliquot of each culture medium was pelleted, resuspended in 460μL Resuspension Solution (200μL 20% glucose, 200μL Tris 25mM pH7.6 10mM EDTA and 60 μL 0.5M EDTA) and lysed by using Lysing Matrix B Tubes (MP Biomedicals) in presence of 500μL acid phenol. RNA was then purified by TRIzol-chloroform extraction, precipitated with isopropanol and washed in 75% ethanol. RNA samples were subjected to two DNase I treatments (TURBO DNase; Ambion), each followed by further phenol-chloroform purification. RNA quality was checked by gel electrophoresis and NanoDrop analysis. First-strand cDNA was synthesized from 5μg total RNA by using *polA*-, *ipaB*-, *virB*- and *virF*-specific primers with a unique 5’ tag sequences not present in *S*. *flexneri* M90T (S3 Table). Reverse transcription was performed in presence of actinomycin and samples were then treated with RNAse H (Life Technologies) at 37°C for 20 minutes. After purification, cDNA was analysed by quantitative real-time PCR with Power SYBR green PCR master mix (Applied Biosystems) using a gene-specific forward primer and a tag-specific reverse primer to ensure strand-specific cDNA amplification. StepOnePlus real-time PCR system was used to monitor the reaction. Results represent the average of four biological replicates and were normalised to *polA* cDNA levels by using the 2-ΔΔCt method [60]. Values are shown in relation to wild type M90T 2-ΔCt levels, which are indicated as 1. Therefore, values less than 1 indicate decreased transcription levels of the target genes in the analysed samples. Statistical analysis was performed using one-way ANOVA with Tukey’s multiple comparison test.

## Supporting information

S1 Table Sequence identity and coverage of ISs involved in PAI deletions. Coverage and identity values were obtained by aligning the two IS copies from each variant by BLASTN. %GC content was calculated for IS and refers to the copy in order of their numbering according to Buchrieser *et al*. [9].
(TIF)

S2 Table Strains used in this study. (TIF)

S3 Table Primers used in this study. (TIFF)

S1 Fig CR binding loss in *S*. *flexneri* M90T and native *mvp*^WT^ at 21°C. (A) Proportion of CR^-^ colonies in *S*. *flexneri* M90T and native *mvp*^*WT*^ (reproduced from Fig 4A for statistical comparison) relative to total colonies after approximately 50 generations of growth at 21°C. Solid line: mean of six biological replicates. (B) Multiplex PCR analysis was performed as described for Fig 1B. Results are shown as mean (n = 6 biological replicates). n.s., not significant; values analysed with one-way ANOVA, Tukey multiple comparisons test.
(TIF)

S2 Fig Plasmid sequences from native *mvp*^WT^ and ectopic *mvp*^WT^ at 21°C. Alignment of plasmid sequences of 10 independent CR^-^ colonies emerging from native *mvp*^WT^ (A) and ectopic *mvp*^WT^ (B) at 21°C which retained their virulence-related genes tested by multiplex PCR. Images were created as described in Fig 1C.
(TIF)

S3 Fig IS1294 copies on pINV and the chromosome. The IS elements were numbered clock-wise, using the origin as a starting point [9].
(TIF)

S4 Fig Example of multiplex PCR gel image of the positive and negative control. Paired adjacent columns correspond to a single assayed colony: pINV +, amplicons originating from *S*. *flexneri* M90T genomic DNA; pINV–, amplicons originating from *S*. *flexneri* BS176 genomic DNA. Each amplified locus is indicated in white letters. The sizes of a kb marker are shown.
(TIF)
