# Supplementary figures and images for: Genetic plasticity of the Shigella virulence plasmid is mediated by intra- and inter-molecular events between insertion sequences
Source: PLoS Genet. 2017 Sep 25;13(9):e1007014. doi: 10.1371/journal.pgen.1007014 (PMC5629016; doi:10.1371/journal.pgen.1007014)

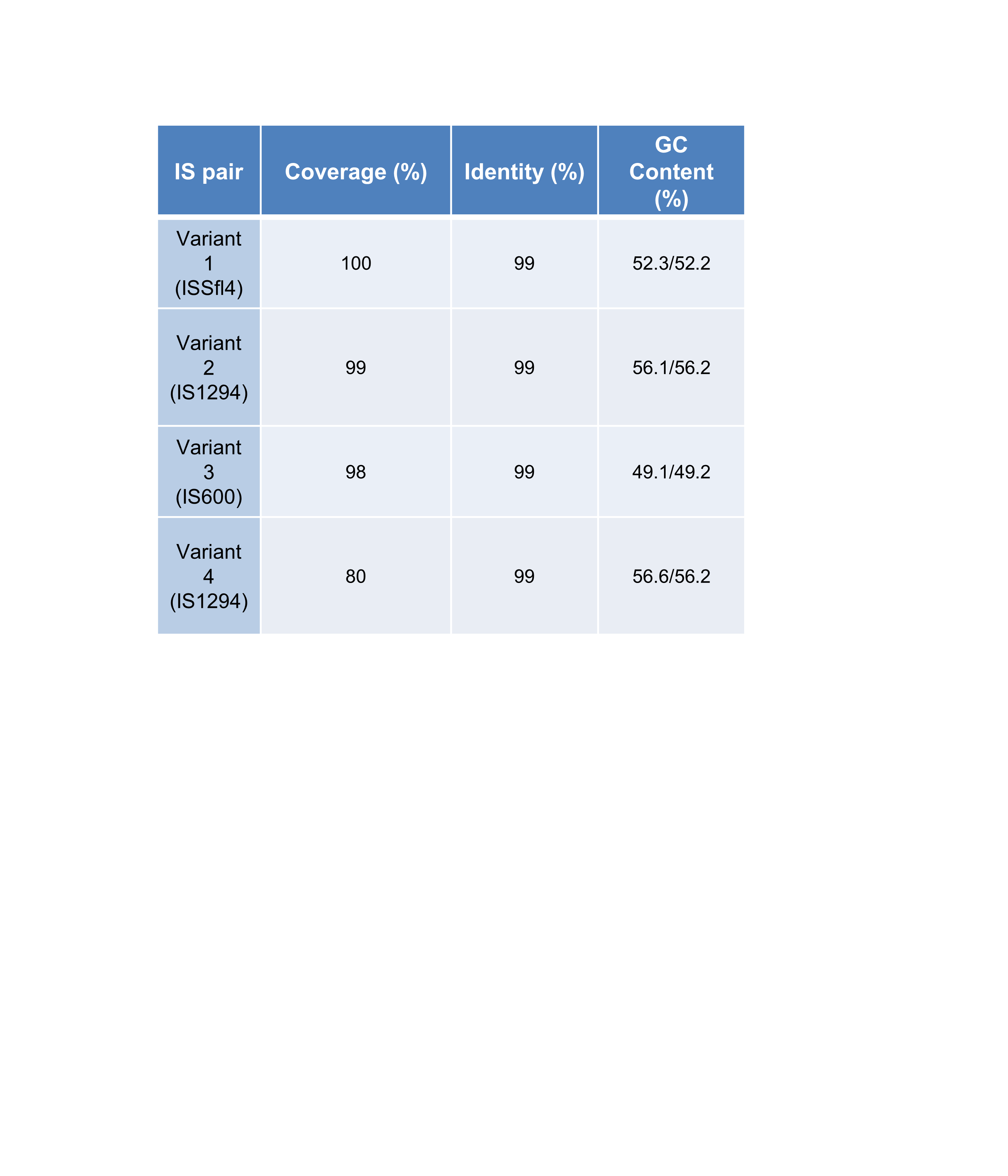

Supplement: S1 Table — Coverage and identity values were obtained by aligning the two IS copies from each variant by BLASTN. %GC content was calculated for IS and refers to the copy in order of their numbering according to Buchrieser et al. [9]. (TIF) [file pgen.1007014.s001.tif]

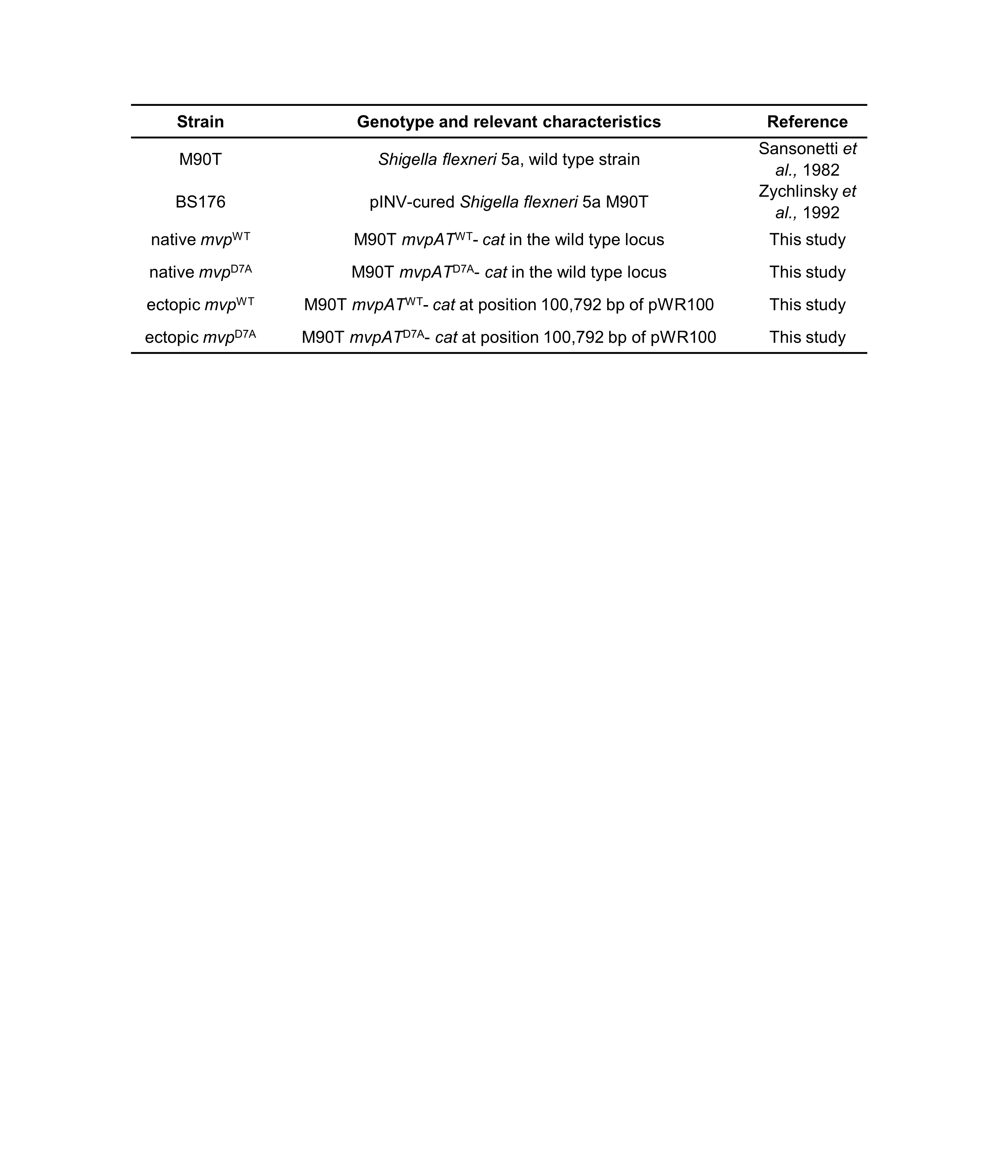

Supplement: S2 Table — (TIF) [file pgen.1007014.s002.tif]

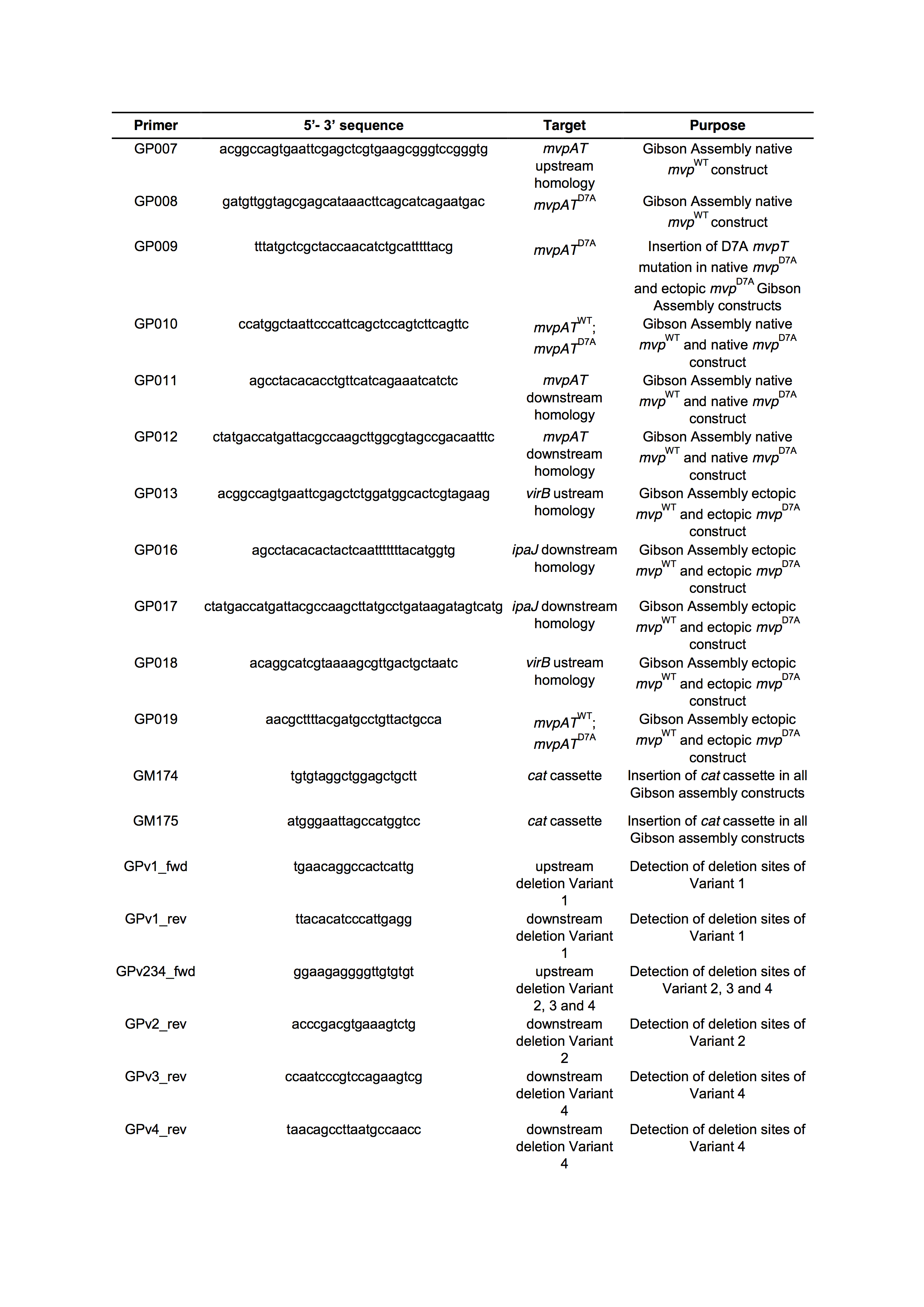

Supplement: S3 Table — (TIFF) [file pgen.1007014.s003.tiff]

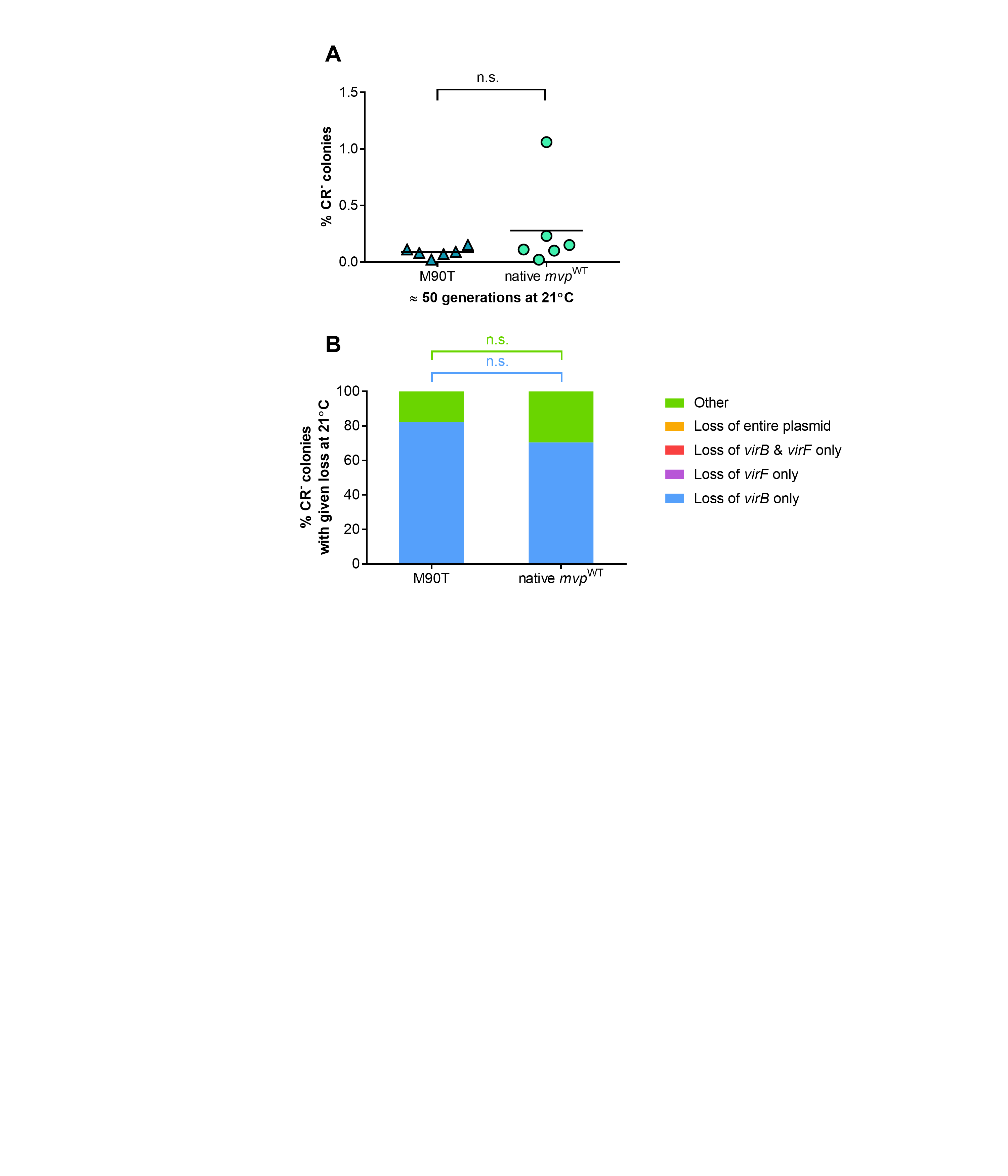

Supplement: S1 Fig — (A) Proportion of CR- colonies in S. flexneri M90T and native mvpWT (reproduced from Fig 4A for statistical comparison) relative to total colonies after approximately 50 generations of growth at 21°C. Solid line: mean of six biological replicates. (B) Multiplex PCR analysis was performed as described for Fig 1B. Results are shown as mean (n = 6 biological replicates). n.s., not significant; values analysed with one-way ANOVA, Tukey multiple comparisons test. (TIF) [file pgen.1007014.s004.tif]

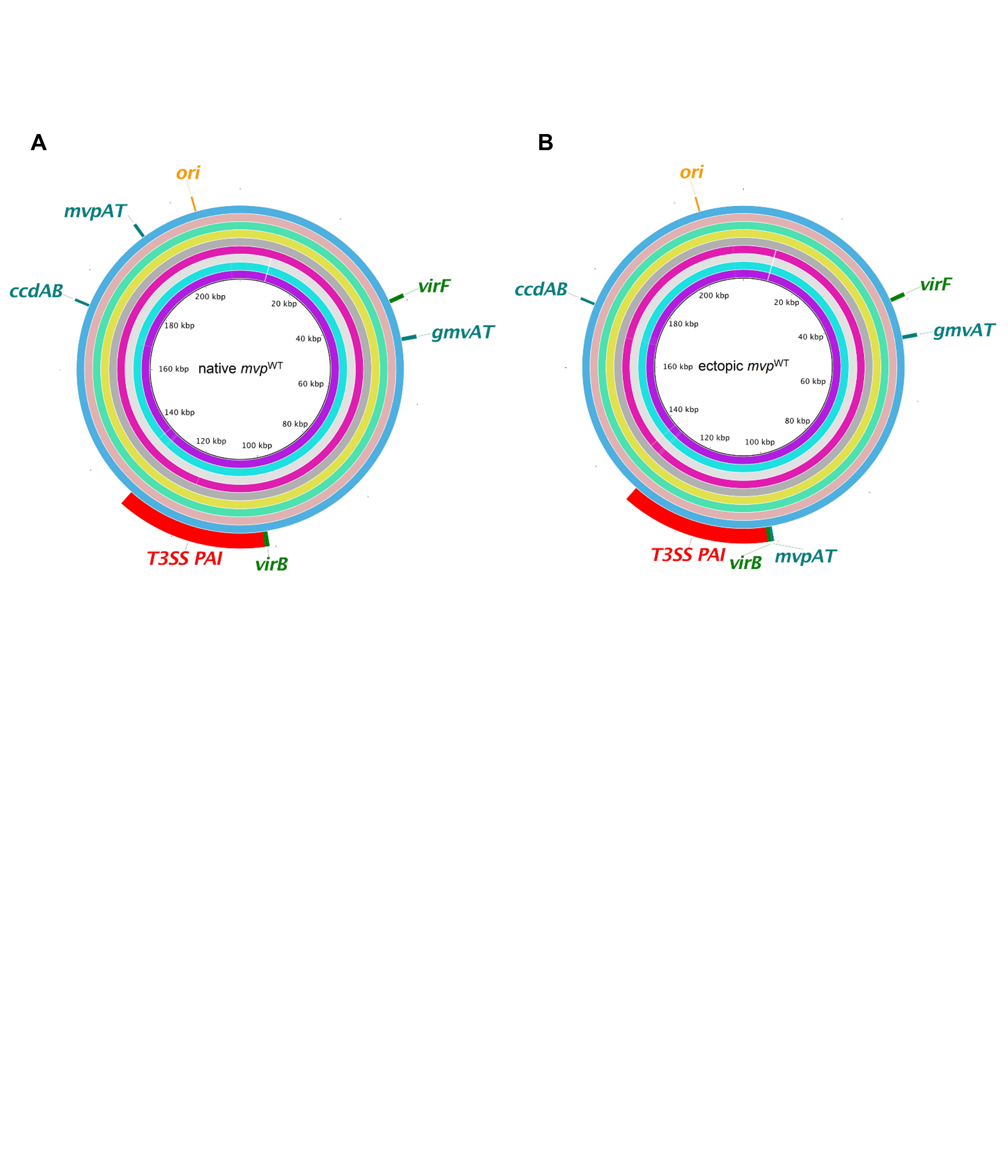

Supplement: S2 Fig — Alignment of plasmid sequences of 10 independent CR- colonies emerging from native mvpWT (A) and ectopic mvpWT (B) at 21°C which retained their virulence-related genes tested by multiplex PCR. Images were created as described in Fig 1C. (TIF) [file pgen.1007014.s005.tif]

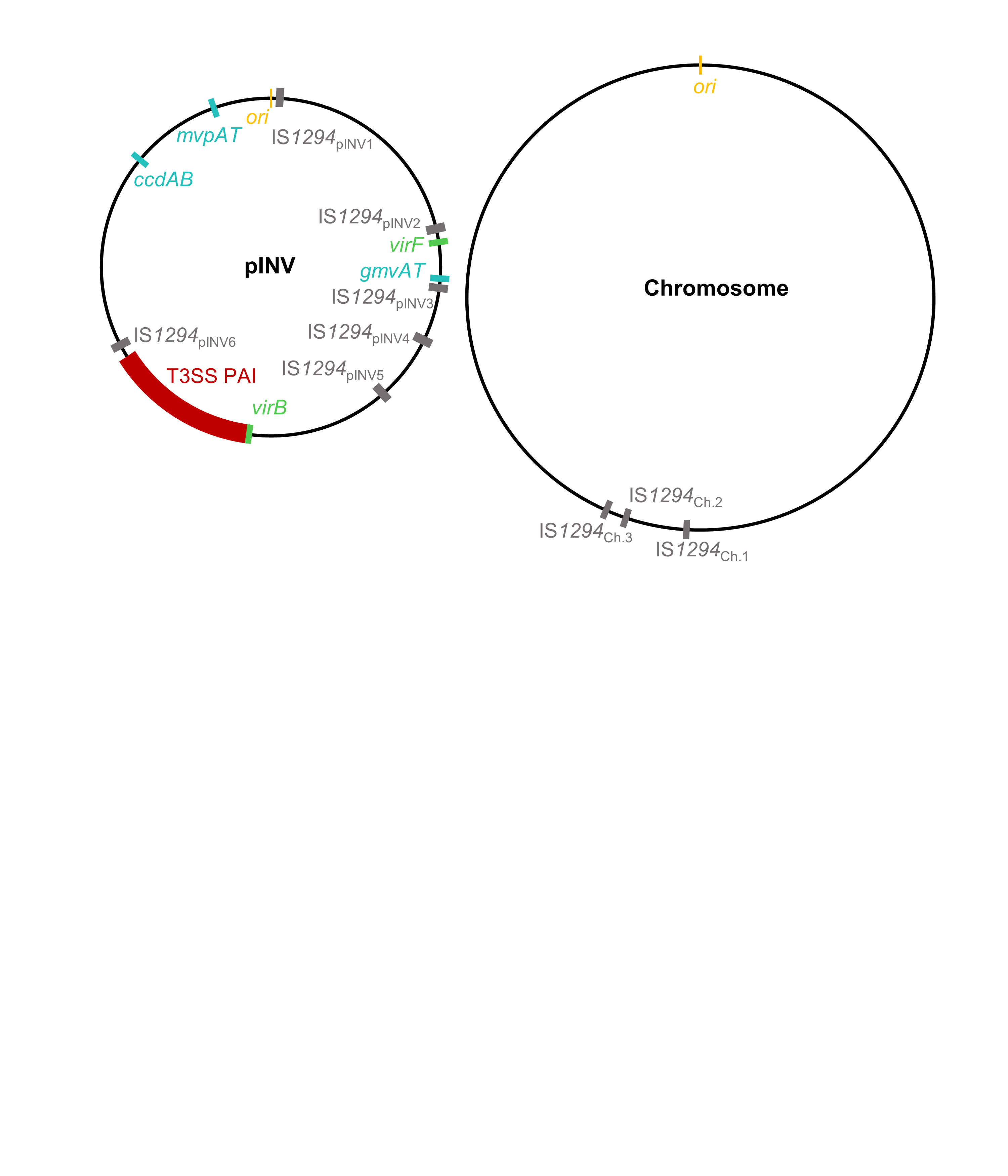

Supplement: S3 Fig — The IS elements were numbered clock-wise, using the origin as a starting point [9]. (TIF) [file pgen.1007014.s006.tif]

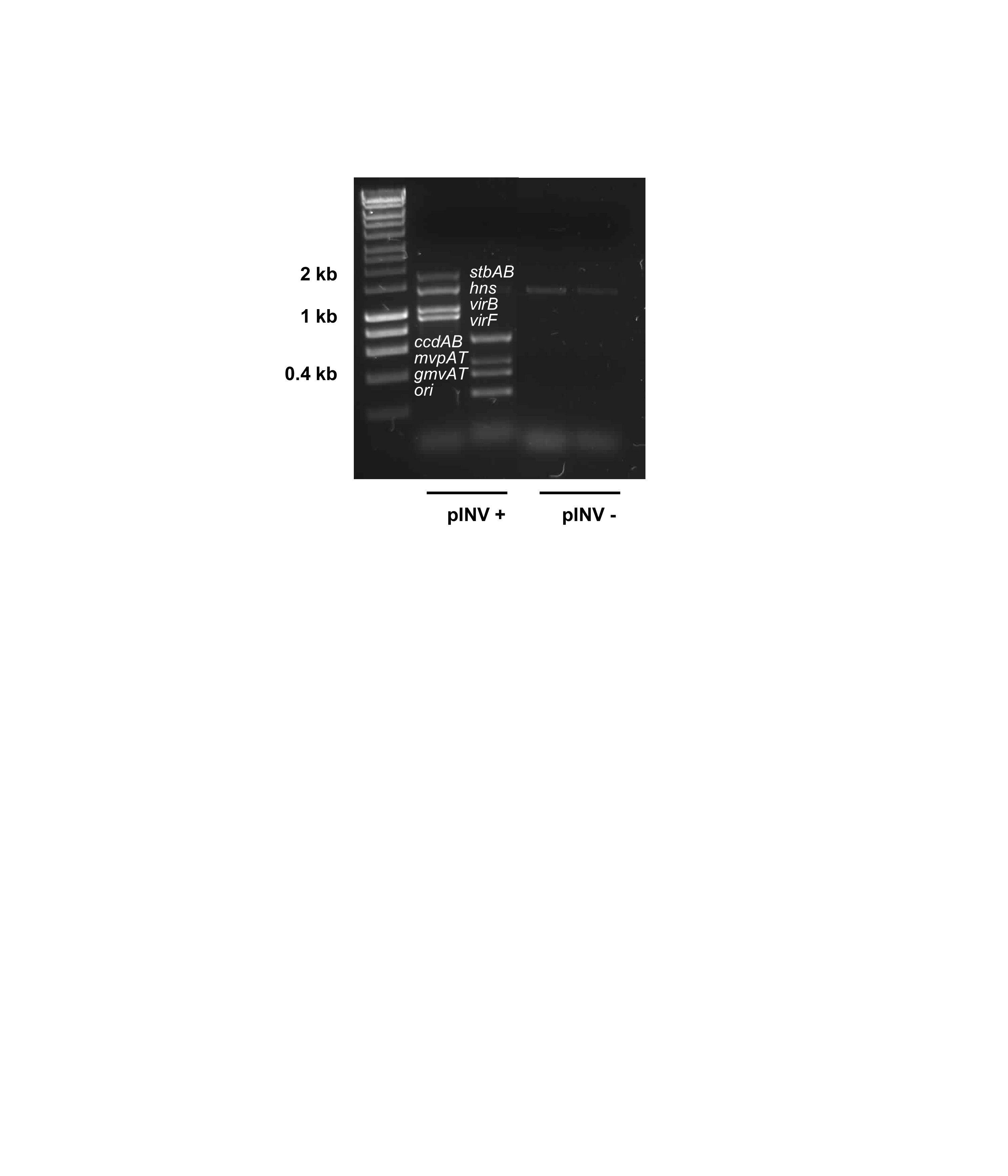

Supplement: S4 Fig — Paired adjacent columns correspond to a single assayed colony: pINV +, amplicons originating from S. flexneri M90T genomic DNA; pINV–, amplicons originating from S. flexneri BS176 genomic DNA. Each amplified locus is indicated in white letters. The sizes of a kb marker are shown. (TIF) [file pgen.1007014.s007.tif]
